# Illustrations of Some of the Forms of Sudden Death

**Published:** 1845-10

**Authors:** D. J. T. Francis


*Illustrations of some of the forms of Sudden Death.* By D. J. T. Francis, M. B.—We invite the attention of all our readers to the following summary of Dr. Francis' very instructive communication, (in Guy's Hospital Reports) and beg of them to bear in mind the remarks which we have made on the treatment of Apoplexy in a previous page. They will find, in the present article, fresh proofs of the necessity of caution in the use of blood-letting in the medical management of a sudden *fit*, whether it be of an apoplectic nature or not.

Within a period of two years and five months, 1000 deaths occurred among the inmates of the Manchester workhouse, in which Dr. Francis is the resident medical officer. Of this number, nineteen were instances of what is usually understood by the term "sudden death;" which it is intended, with one or two exceptions, to apply, in the following narration, to those cases wherein life has become extinct within a quarter of an hour from the commencement of the fatal seizure. We proceed to analyse them; this it is the more easy to do, as they are arranged under separate heads.

I. *Cases in which the Cause of Death was seated in the Brain.*

Three examples are given. The first occurred in a man, æt. 56, who, although he had been disabled for four or five years by a partial palsy of the left arm and leg, continued in good general health. One day, while standing up in the middle of the yard, passing his urine (he had considerable difficulty in micturition,) he was observed to glide easily to the ground: he gave two or three short cries, but there was no struggling. "Within two minutes after he fell, being close at hand, I saw him; but from this time there was no attempt at inspiration, the whole apparatus being quite motionless, although the heart continued to beat, pretty forcibly at first, and afterwards with a series of three or four faint pulsations, and gradually lengthening intervals, for nearly three minutes after I first saw him. The face was not remarkably livid: the pupils were somewhat, but not much, dilated. From these circumstances, an effusion of blood interfering with the respiratory portion of the medulla oblongata, and annihilating the perception of a 'besoin de respirer,' was predicted."

*Dissection.*—Extensive effusion of blood at the base of the brain and around the medulla oblongata. Lateral ventricles empty; a few clots in the fourth ventricle. The arteries at the base were studded with opaque atheromatous spots; on gently throwing a current of water into the basilar artery, two jets issued from apertures in the posterior cerebral arteries. *Heart* sound, or nearly so. Some traces of an old pleuritic and pneumonic attack in the left lung. Abdominal viscera sound.

*Case* 2.—A man, æt. 58, having been absent for a minute in the water-closet, was heard to fall on the gound He was found comatose; the breathing was slow and heavy; the countenance was of a sub-livid hue; the pulsations at the heart and wrist were full and powerful, and continued most distinctly for some time after all attempts at respiration had ceased. The pupils were at first contracted; and afterwards somewhat, but not greatly, dilated. Death took place in about eight minutes. This man had had an attack of partial hemiplegia six years before.

*Dissection.*—The lateral ventricles of the *Brain* contained a large quantity of bloody serum and florid clot. Great softening of the *fornix* and of the ventricular parietes. There was extensive effusion of recent coagulated blood at the base of the brain. The fourth ventricle was filled with a coagulum, which, after firmly investing the medulla oblongata, passed down the spinal canal, as far as it could be seen. There was no appearance of disease in the posterior cerebral or basilar arteries; but, when water was thrown into the latter, it escaped frecly from the vessels in the fissure of Silvius, which were becoming opaque and hardened. *Lungs* almost entirely healthy. Pericardium adherent in nearly its whole extent. Left ventricle of the *heart* much thickened; valves sound. Both kidneys granular.

*Case* 3.—A man, æt. 54, a tailor, soon after eating a hearty breakfast of porridge and milk, and while sitting in the usual cross-legged posture at his work, suddenly complained of pain in the head, and fell from his seat, becoming almost immediately quite insensible. He had returned from the water-closet only a few minutes previously. He died within five minutes from the moment of the seizure.

*Dissection.*—All the sinuses of the *brain* were enormously distended: its substance throughout seemed healthy. Ventricles empty. No appearance of extravasation anywhere. After the brain was removed, several ounces of blood flowed from the spinal canal and the base. Both *lungs* were excessively gorged with fluid blood. The *heart* was small, pale, and flabby; left ventricle thin and lacerable; when cut into, there escaped about four ounces of dark fluid blood, much of which came from the aorta. The right side was not quite so full of blood; the ventricle containing a loose fibrinous clot, the only one found in the body. The abdominal viscera were dark and congested, but seemingly healthy otherwise.

*General Remarks.*—With respect to these three, and such like cases, we may presume that the cause of death is pressure upon the *medulla oblongata*—whether applied immediately to the part from extravasation, or indirectly from the general distension of the cerebra mass with blood—and the consequent suspension of the function o Respiration. The following illustrative observations will be rea with interest.

"Independently of the pathological interest which attaches to these cases, they were alike characterized by a remarkable symptom, which, as it was carefully observed, could not fail to arrest the attention;—I mean, the decided manner in which the action of the heart and the pulse at the wrist were prolonged, after the respiration had completely ceased. This circumstance was obviously due for its cause to the especial manner in which the hæmorrhage that had taken place had compressed the medulla oblongata, and so annihilated the function of the part which presides over the respiratory movements; whilst the heart, being less completely under the influence of that portion of the nervous system, had continued in its action, ceasing only from the obstructed circulation through the lungs. Experiment and previous pathological observation had long since established the fact of the dependence of the respiration upon integrity of the medulla oblongata; but the availability of the priority in cessation of the action of the heart or lungs, as a means of elucidating the causes of sudden death, appeared further to be an object well worthy of careful investigation." 97.

II. *Cases in which there was Congestion of the Right Heart and Lungs.*

*Case* 4.—A woman, æt. 73, was sitting upon the close-stool, to which she had walked unassisted, and suddenly exclaimed that she was blind. She was helped into a chair, and, in less than ten minutes (perhaps five) was dead. There was no struggling; the countenance remained quite placid. She had been heard to complain of palpitation of her heart for two or three days before her death.

*Dissection.*—The *brain* was remarkably healthy; so were the *lungs* upon the whole, except that they were much congested. *Heart* rather large; much fat upon its surface and among its fibres; the valves healthy; all the cavities contained a great quantity of fluid blood like tar: upwards of three pints flowed from the section of the great vessels. There was a remarkable accumulation of fat, and also many enlarged bronchial glands, closely applied around the base of the heart. "The loose cellular tissue about the apex of the pericardium had become converted into a dense steatomatous tumour, which had insinuated itself between the vessels, and left visible marks, in the alteration of form which had followed, of permanent compression of the pulmonary arteries and veins." The parenchymatous viscera of the *abdomen* were much congested.

Here then was an example of genuine Asphyxia.

The next *case* (No. 5) is in many respects similar to the preceding one. An old woman was in the act of dressing herself, when she suddenly became faint, sank into a chair, and in a few minutes expired. She had been complaining, the day before, of dyspnœa.

*Dissection.*—In this case, too, there was an immense mass of enlarged and indurated bronchial glands—one or two of which were almost as large as a hen's egg—surrounding the lower part of the trachea and the bronchi, which were flattened and compressed. The great blood-vessels also must have been subjected to considerable pressure, when the morbid mass was turgid with blood. The *lungs* were saturated with fluid blood. The *heart* was rather large. The head was not examined.

*Case* 6.—An idiot boy, 10 years of age, was found dead in his bed. He had never been affected with epilepsy. On *dissection*, the chief morbid appearance found was the *thymus* gland of very great size, mottled with intense congestion. The *thyroid* also, and the communicating slip between the two lobes, were in a similar condition. When full of blood, they must have caused considerable pressure upon the great vessels and bronchi. The whole venous system was much congested and loaded with blood.

The enlargement of the Thymus in this case deserves notice, from the circumstance that this lesion is believed to have something to with some alarming chest diseases in children.

*Case* 7 is entitled, *Emphysema of the Lungs*, *Fat Heart*, *Repletion*, *Exercise*, *Exposure to Cold; sudden Death within five minutes.* The most conspicuous necroscopic appearance was enormous distension of the cavities of the *heart* with fluid blood: the walls of the ventricle on this side were much thickened. Both *lungs* were emphysematous; their central parts loaded with blood and serum. The *brain* was sound.

*Case* 8 was similar in most respects to the preceding: the patient died within five minutes after the attack.

*Case* 9 was one of sudden Hæmoptysis. A cachectic young man, while carrying a box of medicines up stairs, was seized with a copious exspuation of blood. He died within five minutes of the attack. On dissection, the *lungs* were found to be deeply congested, and had acquired a spotted appearance in parts, from blood having been drawn into the air-cells. The *heart* was sound. The liver, spleen, and kidneys were immensely hypertrophied,* and soft and pale in texture.

*Case* 10 affords another example of Asphyxia from pulmonary hæmorrhage. The patient, æt. 33, was phthisical. While straining and coughing upon the close-stool, he began to spit blood, and continued doing so until his death, in about 12 minutes from the moment of the attack.

*Dissection.*—In the upper lobe of the left lung was a large irregular vomica, from which the hæmorrhage appeared to have taken place; for along its wall ran a branch of the pulmonary artery, almost entirely denuded of surrounding tissue. Water thrown into the pulmonary artery escaped abundantly into this vomica, and apparently from the denuded vessel, where it was dipping into the pulmonary tissue; but the existence of an opening in it could not satisfactorily be ascertained. In several parts the blood appeared to have been drawn into the areolar tissue of the lung, small patches of increased redness and density being scattered about. Heart healthy.

* This term is greatly misused in the present day. The viscera mentioned were doubtless much enlarged in dimensions; but surely the enlargement was not from *over-nutrition*.

*Case* 11 is entitled *Hæmoptysis—Obstructive Disease of the Heart —Sudden Death in the Night.*—The patient was found dead in her bed. She seemed to be quite well the night before. On *dissection*, blood was found throughout the air-passages generally. The *lungs* were greatly congested. The mitral valve was much puckered, and the left auriculo-ventricular opening considerably contracted.

In the three preceding cases, death seems to have been occasioned by the obstruction to the admission of air into the pulmonary cells from the extravasation of blood. "The chief danger, under such circumstances, would seem to arise from the suddenness and extent of the hæmorrhage, the blood being poured out more rapidly than its evacuation can be effected ; in addition to which, it coagulates about the fauces and mouth, especially when the expulsive efforts on the part of the patient begin to fail. When these conditions are most effectually in operation, asphyxia is accomplished as speedily as in cases of drowning, strangulation, and the like ; as was evidenced in Case 9, where life became extinct in from three to four minutes from the moment of seizure."

III. *Cases in which Death was the result of Syncope.*

Five examples are given under this head. In the first the death was almost instantaneous from *rupture of the left auricle.* The pericardium contained nearly 20 oz. of blood.

The next case is more interesting from the more gradual sinking of the patient, who was 59 years of age. Dr. Francis says :

"At 10 A.M. on September 22d, 1844, I was sent for to visit him. He had been taking a short walk, and whilst in the act of practising upon the violincello, previous to playing at church, he was seized with intense pain across the forehead, faintness, and vomiting. He was sitting with his body bent forwards : the face was pale, shrunk, and expressive of agony : the pupils very slightly dilated. The intellect was unimpaired : the heart's action was very faintly perceived; the pulse scarcely at all.

" He was conveyed to bed, but died in about one hour and a half from the commencement of the seizure, having previously experienced some severe pains in the cardiac region." 89.

*Dissection.*—The pericardium contained about 26 oz. of blood. *Heart* not much affected ; but the aorta greatly diseased. Its internal coats were very brittle, and lacerable. At one point there was a round, ragged, opening in the external coat, large enough to admit a crow-quill ; and about half an inch lower down there was a longitudinal slit in the internal coat of the vessel. Thus the lacerations in the two tunics did not correspond. Hence the cause of the extravasation of blood into the pericardial sac having been slow and gradual. The circumstance of this patient having been suddenly seized with " intense pain across the forehead, faintness, and vomiting," might naturally have suggested the idea that it was a cerebral attack. Perhaps it was so in part; for, although the substance of the brain appeared, on dissection, to be quite sound, its arteries were extensively ossified, so as in some parts to be nearly obliterated.

In *Case* 16, the sudden death was owing to the *rupture of an Aneurism* of the descending thoracic aorta into the left pleura. The heart was sound. The other two cases under this head need scarcely be mentioned. In one there was a large aortic aneurism; the patient died almost immediately after attempting his own life, although he inflicted scarcely any injury upon himself.

### IV. *Cases where Death resulted from Epilepsy.*

Three examples are given. In two, the sudden death occurred during the night in bed. In the third the patient had had several fits during the day of her decease: after one, at 9 P.M. being at the time in bed, deep coma supervened, and she died in a few minutes after its commencement. In none of these cases did dissection reveal any thing conclusive as to the cause of death. How unsatisfactory, upon the whole is the necroscopic history of this disease, Epilepsy!

Here we close our notice of this valuable paper, with the following interesting extract. "In the absence of previous history, and under circumstances which afford but little time for deliberate reflection, the observation of the condition of the pulse and breathing would seem, so far as the few cases herein cited will allow, to be available with readiness in the diagnosis of the causes of these sudden deaths: at the least, encouragement is given for further investigation. It has fallen within the experience of most persons to have met with such cases, where, in the absence of post-mortem examination, various interpretations have been put upon the symptoms, whether referrible to lesion in the head or chest, by different observers who have been present. Thus the sudden approach and ingravescence of the attack are not at all times in favor of the heart, as is borne out by the three cerebral cases, especially Case 3. A pallid or livid face may be compatible with most of the varieties of sudden death; and in those cases wherein the state of the pupils is noticed, no uniformity was observable. Again, intense pain in the head, which, if present, might seem fairly to indicate a cerebral lesion, was most strikingly evidenced in the case of I—— (Case 13), who died from hæmorrhage into the pericardium. Vomiting, also was a prominent symptom at the advent of this man's seizure. In fact, most of these signs are indicative merely of certain accidental complications not necessarily connected with the fatal lesion; and may therefore exist with lesions of totally different characters, provided only that the conditions upon which they depend happen to be present."

Our readers will probably have observed, from the preceding cases, how frequently the fatal attack appeared to be owing to the straining that attends the act of defæcation.—*Medico-Chirurgical Review.*

